# Who Gets Mental Health Care? The Role of Burden and Cash-Paying Markets

**DOI:** 10.1001/jamahealthforum.2024.0210

**Published:** 2024-03-01

**Authors:** Julie M. Donohue, Jennifer Leah Goetz, Zirui Song

**Affiliations:** 1, Department of Health Policy and Management, University of Pittsburgh School of Public Health; 2. McLean Hospital, Harvard Medical School; 3. Department of Health Care Policy, Harvard Medical School; 4. Department of Medicine, Massachusetts General Hospital; 5. Center for Primary Care, Harvard Medical School

Long-standing inequities in access to mental health care persist. Only 47% of adults with mental illness received any mental health services in 2021,^1^ due in part to workforce shortages. More than half of US residents live in a mental health professional shortage area,^2^ and more than half of all US counties lack a psychiatrist, with rural areas most affected.^3^ The supply of child and adolescent psychiatrists and geriatric psychiatrists is even more limited.^3^

However, these numbers do not tell the whole story: access is also poor for those who need to use their health insurance to pay for care, which includes most individuals, and nearly half of psychiatrists do not accept insurance. In 2010, for example, 45% of psychiatrists did not accept insurance, a share much higher than most other physician specialties.^4^ Moreover, accepting insurance does not necessarily mean devoting substantial time to serving insured patients. A 2013 study from Massachusetts,^5^ which has higher psychiatrist insurance participation than the US overall, found that while 79% of psychiatrists participated in at least 1 insurance network, only 6% billed insurance for a substantial volume of patients (at least 300 patients per year).

Access to psychiatrists in Medicaid, which has lower reimbursement rates, is particularly limited. Psychiatrists are more than twice as likely to accept new self-pay patients than they are to accept new Medicaid patients.^6^ Importantly, people with serious mental illnesses are overwhelmingly insured by public sources. For example, 70% of individuals with schizophrenia are enrolled in Medicaid.^7^ The number of US medical school graduates entering psychiatry residencies has increased during the past 12 consecutive years.^8^ However, a substantial share of graduates go into private practice and may not accept insurance, rendering access to their care dependent on patients’ socioeconomic means.

## Role of Administrative Burden

In this issue of *JAMA Health Forum*, Zhu and Eisenberg^9^ evaluate a plausible barrier to psychiatrists and other mental health clinicians joining insurance networks: administrative burden. They elucidate administrative barriers and frictions all along the care continuum, from difficulties becoming credentialled by insurance plans to time costs and direct financial losses associated with byzantine claims processing systems. Lengthy requirements for clinicians at new patient intake can delay care. Zhu and Eisenberg^9^ rightly point out that mental health clinicians often navigate this administrative complexity on their own because they are often operating in solo or small group practices with no administrative staff.

These burdens are real and costly to clinicians. In a world where “time is money,” dealing with our nation’s fragmented, administratively burdensome insurance system—joining insurer networks, negotiating payment rates, managing prior authorizations, and payment denials—not only diminishes the joys of clinical practice, but also saps financial opportunity. This is particularly salient for specialties with relatively low infrastructure costs, including psychiatry, that have an alternative—a robust private market of willing, cash-paying clients.

## Role of the Private Cash-Paying Market

That nearly half of psychiatrists do not accept insurance is not a new phenomenon. Low participation rates predate our modern insurance system, characterized by narrow networks and utilization management burdens. A 1978 study conducted by the American Psychiatric Association^10^ reported that fully 51% of payments for private practice psychiatrists were from private (ie, out-of-pocket) resources, while 46% came from private insurance, and 3% from government insurance. Acceptance of cash payments has been common among psychiatrists in private practice settings, existing in one form or another for well over 100 years. Private (often solo) practice undertaken as a full-time or part-time activity by psychiatrists employed by hospitals, health systems, or publicly funded clinics has been critical to the financial viability of the profession.^11^

For decades, however, studies have commented on the inequities likely to result from a system highly reliant on cash payments from patients. A 1956 article in the *American Journal of Psychiatry* commented that the prosperity of private practice psychiatrists concealed “a curious economic paradox” that “the private practitioner of psychiatry cannot afford to work for what the average patient can afford to pay.”^12^ Similarly, a 1975 review on the readiness of psychiatry for national health insurance in the US concluded that “since the more expensive treatments, such as psychoanalysis, are used disproportionately by less ill patients with higher incomes, the poor would be disproportionately taxed to subsidize the rich.”^13^

Despite expansions in insurance coverage and legal mandates that health plans include mental health care coverage, out-of-pocket costs remain a major barrier. In 2021, among patients who needed mental health care but who did not receive it, 55% of people with serious mental illness said they could not afford the cost, and an additional 19% and 14% cited inadequate insurance payment and coverage for care as key barriers, respectively.^1^ In addition to disparities based on ability to pay are racial and ethnic disparities. In 2021, 52% of White adults with mental illness received any treatment, whereas Black, Hispanic, and Asian adults with mental illness reported treatment rates of 39%, 36%, and 25%, respectively.^6^

## Toward More Equitable Access

The tradition of private practice psychiatry coupled with the availability of cash-paying patients exacerbates mental health workforce shortages, especially when demand outstrips supply. Reductions in administrative burdens would help. However, these reductions alone probably would not solve the access problem. Money is needed. The empirical question that remains unanswered is how much. In areas where 1 hour of private practice yields cash payments of $500 to $1000—but even generous private insurance offers $100,^14^ with even lower payments by Medicare or Medicaid—the price gap seems so wide that it borders on insurmountable. Even if enough of society’s resources were invested to double Medicaid, Medicare, or commercial prices, the private market could remain advantageous for many psychiatrists.

There is probably not enough money to equalize the playing field. Higher prices for care are ultimately financed by wages or taxes in the US. Yet neither wages nor tax revenues are in excess supply. For employers, especially large employers that are self-insured, paying more for their workers’ health care requires taking from profits or wages. For state Medicaid agencies, paying more for mental health means less money for other state programs. For federal programs including Medicare, the trade-off involves taxes or debt, if not lower federal outlays for other needs.

For ease of illustration—if mental and behavioral health care is 10% of total health care spending—doubling insured prices for mental and behavioral health care (without any volume response) means a 10% increase in total health care spending. That is a lot. Even then, the private-pay market might still win.

## Is There a Path for Coming Back (to Insured Patients)?

Despite this uphill battle, innovation, experimentation, and multisector partnerships to improve access are urgently needed. First, state Medicaid agencies could experiment with increasing payment rates to psychiatrists and other mental health professionals combined with reducing administrative burden as suggested by Zhu and Eisenberg.^9^ The US Centers for Medicare & Medicaid could encourage states and managed care organizations to experiment with combinations of changes to *both* financial incentives and administrative simplifications, with the explicit goal of bringing more mental health professionals into public and private insurance networks.

Second, academic medical centers that self-insure their own employees could experiment with incentive programs. In many centers that employ psychiatrists, psychologists, and other mental health clinicians as faculty, the institution’s own employees (from lower to higher wage workers) lack access to mental health care despite having employer-sponsored insurance—in part due to their colleagues seeing patients in private practice during a substantial part of the week. Could rewards—eg, new professional recognition or academic opportunities—help attract these colleagues’ needed skills back into insurance networks, starting with their fellow employees?

Third, at least 1 US county has implemented a generous loan-repayment program (up to $45 000) for mental health professionals who commit to practicing in publicly funded behavioral health service organizations.^15^ This program leverages partnerships with local educational institutions, foundations, and state agencies. With support from state and local governments and universities, other jurisdictions could follow. These efforts could take many forms and their effect on access to mental health care should be evaluated.

For more than a century, *if you build it, they will come* has symbolized the private-pay market for mental health care. Yet most patients do not get to participate. So rather than taking the private market away, the challenge to policymakers is changing the rules to create a more level playing field. Only then will the US be able to address the burden of untreated mental illness.

## References

[1] Substance Abuse and Mental Health Services Administration. Key substance use and mental health indicators in the United States: Results from the 2021 National Survey on Drug Use and Health (HHS Publication No. PEP22-07-01-005, NSDUH Series H-57).. Accessed January 19, 2024. https://www.samhsa.gov/data/sites/default/files/reports/rpt39443/2021NSDUHFFRRev010323.pdf

[2] Health Resources & Services Administration. Health Workforce Shortage Areas Dashboard. . Accessed January 19, 2024. https://data.hrsa.gov/topics/health-workforce/shortage-areas

[3] University of Michigan Behavioral Health Workforce Research Center. Estimating the Distribution of the U.S. Psychiatric Subspecialist Workforce. Accessed January 19, 2024. https://www.healthworkforceta.org/wp-content/uploads/2023/07/BHWRC_Estimating-Distribution-of-US-Psychiatrist.pdf

[4] Bishop TF, Press MJ, Keyhani S, Pincus HA. Acceptance of Insurance by Psychiatrists and the Implications for Access to Mental Health Care. JAMA Psychiatry. 2014;71(2):176–181. 24337499 10.1001/jamapsychiatry.2013.2862 PMC3967759

[5] Benson NM, Myong C, Newhouse JP, V F, Hsu J. Psychiatrist participation in private health insurance markets: paucity in the land of plenty. Psychiatric Services Psychiatric Services. 2020;71:1232–1238. 32811283 10.1176/appi.ps.202000022 PMC7708395

[6] Glied S, Aguilar K. The Behavioral Health Workforce Shortage: Can we Make Better Use of the Providers We Have?. Accessed January 19, 2024. https://www.brookings.edu/wp-content/uploads/2023/04/Glied-and-Aguilar-Workforce-Paper-1.pdf

[7] Geissler KH, Ericson KM, Simon GE, Qian J, Zeber JE. Differences in Insurance Coverage for Individuals With Schizophrenia After Implementation of the Patient Protection and Affordable Care Act. . JAMA Psychiatry. 2023;80(3):278–279. 36652234 10.1001/jamapsychiatry.2022.4628 PMC9857749

[8] Moran M Psychiatry Match Numbers Increase for 12th Consecutive Year. 10.1176/appi.pn.2023.05.5.043

[9] Zhu J, Eisenberg M. Administrative frictions and the mental health workforce. JAMA Health Forum. 2024; 10.1001/jamahealthforum.2024.0207 PMC11203202 38517421

[10] Langsley DG. Comparing clinic and private practice of psychiatry. American Journal of Psychiatry 1978;135(6):702–206. 655281 10.1176/ajp.135.6.702

[11] Blain D Private Practice of Psychiatry. Annals of the American Academy of Political and Social Science. 1953;286(1):136–149.

[12] DAvidson HA. The structure of private practice in psychiatry. Am J Psychiatry. 1956;113(1):41–44. 13327070 10.1176/ajp.113.1.41

[13] Frank JD. Review of Psychiatrists and their patients: A national study of private office practice and Varieties of psychotherapeutic experience: Multivariate analyses of patients’ and therapists’ reports. American Journal of Orthopsychiatry. 1976:550.

[14] Benson NM, Song Z. Prices And Cost Sharing For Psychotherapy In Network Versus Out Of Network In The United States. Health Aff. 2020;39(7):1210–1218. 10.1377/hlthaff.2019.01468 PMC8128060 32634359

[15] Allegheny County Department of Human Services. Behavioral Health Fellows Program. Accessed January 19, 2024. https://www.alleghenycounty.us/Government/Employment/Job-Opportunities-by-Department/Human-Services-Careers/Professional-Opportunities/BH-Fellows-Program

